# Persistent pods of the tree Acacia caven: a natural refuge for diverse insects including Bruchid beetles and the parasitoids Trichogrammatidae, Pteromalidae and Eulophidae

**DOI:** 10.1673/1536-2442(2006)6[1:PPOTTA]2.0.CO;2

**Published:** 2006-06-12

**Authors:** D. Rojas-Rousse

**Affiliations:** Institut de Recherche sur la Biologie de l'Insecte, UMR CNRS 6035, Faculté des Sciences, F-37200 Tours, France

**Keywords:** *Pseudopachymeria spinipes*, *Stator furcatus*, *Uscana espinae*, *Monoksa dorsiplana* (Boucek), Horismenus spp, *Dendroctonus* sp, *Camponotus* sp, gregarious parasitoids

## Abstract

The persistent pods of the tree, Acacia caven that do not fall from the tree provide opportunities for the appearance of a diverse group of insects the following season. Such pods collected during the spring of 1999 in Chile were indehiscent with highly sclerified pod walls. In contrast, persistent pods collected in Uruguay after a wet winter and spring (2002) were partially dehiscent, inducing the deterioration of the woody pods, and consequently exposing the seeds. These persistent pods are a natural refuge for insect species, namely two bruchid beetles (Pseudopachymeria spinipes, Stator furcatus), one scolytidae (Dendroctonus sp), lepidopterous larvae, ant colonies (Camponotus sp), one species of oophagous parasitoid (Uscana espinae group *senex*), the gregarious larval-pupae parasitoid Monoksa dorsiplana (Pteromalidae) and two species of Horismenus *spp.*(Eulophidae). The patriline of M. dorsiplana is frequently formed by 1 son + 7 daughters.

## Introduction

Acacia caven (Mol.), a Leguminosae belonging to the sub-family Mimosoideae, is a tree of extra-tropical South America, found between 36°N and 18°S and from the Atlantic to the Pacific. It is usually found in the continental climate of the Grand Chaco region of northern Argentina, central Paraguay and southern Bolivia, as well as in the Mediterranean-type climate zone of Chile, a few parts of southern Brazil and part of Uruguay, where it is often thoroughly integrated with various species of subgenera of Acacia, Prosopis and Schinopsis (Aronson and Ovalle, 1989). A. caven is colloquially known as “espino” or “espinillo”, and is a prominent woody invader in over-grazed cow pastures or abandoned fields. It seems that the espinales are a recent phenomenon in the central valley of Chile, i.e. no more than 500 – 2000 years, probably introduced from northern Argentina by guanacos and pack animals, such as llamas, in Trans-Andean caravans (Ovalle et al.; 1990, Muñoz et al. 1993). A. caven is one of the most specialized members of the legume subfamily Mimosoidea, by virtue of its large colporate pollen, diploid chromosomes, spinescent stipules, and multiseriate, indehiscent pod construction. It is related to, and often confused with, the pantropical Acacia farnesia (L.) Willd., which is not found in Chile (Ovalle et al. 1990). Its phenological behavior is distinctly out of phase with that of all woody plants native to this Mediterranean-climate area. The 'reversed' phenological pattern exhibited by A. caven in Chile was probably a relictual development, which evolved in harmony with the climatic conditions prevailing in the subtropical, dry winter climate of the Chaco, prior to its migration to central Chile in Quaternary times (Aranson et al. 1994). A. caven loses its leaves at the beginning of the rainy season and puts out new leaves at the beginning of the dry season. It flowers in the spring before the appearance of the leaves, which develop completely during the summer. The pods ripen in autumn (Muñoz et al. 1993). The majority of indehiscent pods fall to the ground, but some remain on the trees for several months.

Larvae of the bruchid Pseudopachymeria spinipes feed mostly on the seeds of Acacia (Miller). Although P. spinipes has been imported “into various parts of the Old World, its apparent original distribution was in Argentina, Brazil, Peru and Ecuador” (Johnson and Siemens, 1997). It is also found in Chile where its only host is A. caven (Barriga Tuñon, 1990).

In Chile, analysis of phenological relations between the cycle of A. caven and that of P. spinipes shows that one generation of P. spinipes develops within 10–11 months. Adult longevity is about 20 days, and females oviposit on developing pods, eggs being laid aggregated (Saiz et al., 1977; Avendaño and Saiz, 1978; Saiz et al., 1980). The new larvae have very hard chitinized mouthparts that allow them to enter the developing pods (Avendaño and Saiz, 1978; Saiz et al., 1980). The newly hatched adults mate immediately after emergence (sometimes even before they leave the pod), and then disperse to search for appropriate pods for oviposition. However, as egg-laying behavior is inhibited by the presence of flowers on the host plant and is triggered by the presence of pods (Yates et al., 1989), the females oviposit on the persistent pods of the previous season that have not fallen. These pods allow a high level of bruchids to appear during the following season (Saiz, 1993).

Currently, the only known natural enemies of P. spinipes are a microhymenopterous parasitoid mentioned in a study on agronomical bruchidae pests in Chile (Barriga-Tuñon, 1990), and an oophagous parasitoid from the genus Uscana collected once near Santiago (1981) and once in 1995 near Cauquenes (VII region), identified as Uscana espinae (Pintureau and Gerding) group *senex* (Pintureau et al., 1999).

As persistent pods of A. caven provide a refuge for the population of P. spinipes (Saiz, 1993), we investigated the presence of this species in the persistent pods, as well as the presence of other insects liable to compete with or to parasite P. spinipes. Pods were collected in Chile in November 1997 (region VIII) and in November 2002 in Uruguay (Prado Pare, Montevideo).

## Materials and Methods

### Biodiversity of insects living in persistent pods of A. caven

Ripe pods of A. caven persisting on trees were collected during the spring (November) of 1997 (Chile: region VIII, around the town of Concepcion) and 2002 (Uruguay: Prado Parc, in the city of Montevideo). Each pod was labeled with its place of origin and date of collection, measured (length in cm), and placed in a box covered with a thin cloth. Boxes were stored in a climatic chamber with a 12h/12h photoperiod and a 30°/20°C thermoperiod.

Holes pierced by bruchid beetles were observed on the pod integument, and adult bruchid beetles and parasitoids were counted during a two-month period. Thereafter, each pod was opened to count the number of seeds, and the seeds were kept to count any remaining emergent insects.

### Dynamic Population of Bruchidae and parasitoids developed in one persistent pod

As only one P. spinipes adult can develop in a single seed, the newly emerged population of bruchid beetles corresponds to the number of holes in the seeds, rather than the number of holes in the pods because more than one beetle can use the same hole to escape the pod (Saiz et al., 1977). If only the holes pierced on the pods are counted, the size of the population will be underestimated (Saiz et al., 1977). The difference between the total number of seeds in each pod and the number of seeds attacked corresponds to the quantity of seeds able to start growth and allow the spread of A. coven.

No parasitoid emergence holes were observed on the pods. However, after the rainy season, some of the pods were partially dehiscent, allowing parasitoid adults to leave the pod easily. They could also have passed through the holes pierced on the pod's integument by the bruchid beetles. As only one P. spinipes adult can develop from a single seed, the level of parasitism is apparent from the number of parasitoid emergence holes observed on the seeds.

### Gregarious behavior of Monoksa dorsiplana

The Pteromalidae Monoksa dorsiplana (Boucek) was first described in 1990 in pods of Acacia farnesiana collected in Israel (Boucek, 1990). In order to study some of its life history traits, its development on a substitution host was carried out successfully in the laboratory. The mass rearing of M. dorsiplana was carried out on Callosobruchus maculatus (Bruchidae) reared on Vigna radiata (Leguminosae). The hosts and parasitoids were mass-reared under conditions close to those of their zone of origin: 30°/20°C, 12:12-h L:D, and 70% RH, with synchronous photo- and thermo-periods. Rearing of the Eulophidae Horismenus *spp.* in the laboratory was not carried out successfully.

The offspring of 63 inseminated M. dorsiplana females were studied. Newly emerged females were individually placed with 2 males in Petri dishes for 24h to ensure mating. Females were then individually provided with one V. radiata seed containing either an L4 larva or pupa of C. maculatus (Huignard et al., 1985). These females were given a new host every day until they were 5 days old. Every 24 hours, the seed offered to the female was removed and placed in an Eppendorf tube closed with a cotton cap until the offspring appeared.

## Results

### Biodiversity of the entomofauna associated with the persistent pods

Only Pseudopachymeria spinipes (Erickson) emerged from pods collected in Chile, while the presence of Stator furcatus (Johnson) was also observed in those from Uruguay (identification by Clarence Dan Johnson of Northern Arizona University, USA). Some of the 43 pods collected in Uruguay after the rainy season contained other insect families. The pod apertures facilitated observation of the presence of adults and larvae of the scolyte Dendroctonus sp in the woody walls of the pods, and also lepidopterous larvae feeding on the seeds. In three pods, small nests of Camponotus sp. (Formicidae) were found (identified by Jean Luc Mercier, LEPCO, Tours, France).

Oophagous and larval-pupal parasitoids were also collected. P. spinipes eggs were parasitized by Uscana espinae group *senex* (Pintureau and Gering) (Pintureau et al., 1999), and the larvae were parasitized by Monoksa dorsiplana (Boucek) (Pteromalidae: identified by J. Yves Rasplus, INRA and Gérard Delvare, CIRAD, Montpellier, France) and by two Horismenus spp. (Eulophidae: identified by John Lasalle, Natural History Museum of London and Gérard Delvare, CIRAD, Montpellier, France).

### Pod size and quantity of seeds in a persistent pod

**Figure 1.**
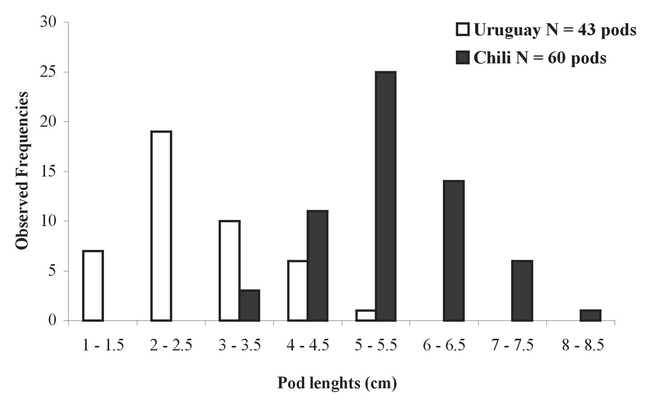
Pod lengths of persistent Acacia caven collected during the spring in Chili and Uruguay.

**Figure 2.**
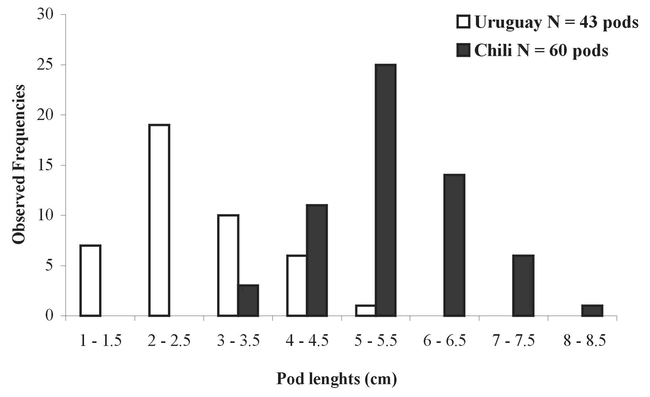
Relationship between the pod length and the number of seeds per pod in Acacia caven.

The pod length (cm) distribution was bimodal according to origin (Chile or Uruguay) (Figure 1). The length of pods collected in Uruguay varied from 1 to 5.5cm (modal class: 2 – 2.5cm) (N = 43: mean length ± SEM = 2.7± 0.3 cm), while those collected in Chile ranged from 3 to 8.5cm (modal class: 5 – 5.5cm) (N = 60: mean length ± SEM = 5.4 ± 0.3 cm) (Figure 1), i.e. twice as long as the Uruguayan pods (ANOVA: XLStats.6 for Windows: F_101,102_ = 167.04, P < 0.0001). The linear regression between pod length and the quantity of seeds per pod was positive: only a couple of values (out of 103) were outside the second confidence interval (Simple linear regression XLStats.6 for Windows: R = 0.62) (Figure 2).

### Intensity of attack by the Bruchidae Pseudopachymeria spinipes and Stator furcatus: Pods collected in Chile

Around 68% of indehiscent pods (41/60) were attacked by P. spinipes, as shown by the presence of 1 to 5 emergence holes (Table 1).

**Table 1.**
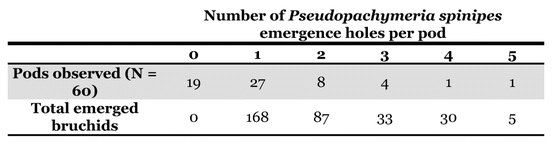
Intensity of attack by Pseudopachymeria spinipes on persistent pods of Acacia caven.

On the majority of attacked pods, we found only one P. spinipes emergence hole (27/41= 66%), and only 2.5% pods had 4 or 5 holes (1/41= 2.5%) (Table 1). As only one P. spinipes adult can develop in a seed and the number of attacked seeds was greater than the number of emergence holes in the pods, we can infer that the majority of bruchids left the pod by the first hole(s) pierced (Table 1).

### Pods collected in Uruguay

The bruchid beetles in pods collected after the rainy season at the end of November 2002 might have been able to leave the partially dehiscent pods without having to pierce the integument. The bruchid beetle attack was therefore analyzed by counting the emergence holes on the seeds of 50 pods.

**Figure 3.**
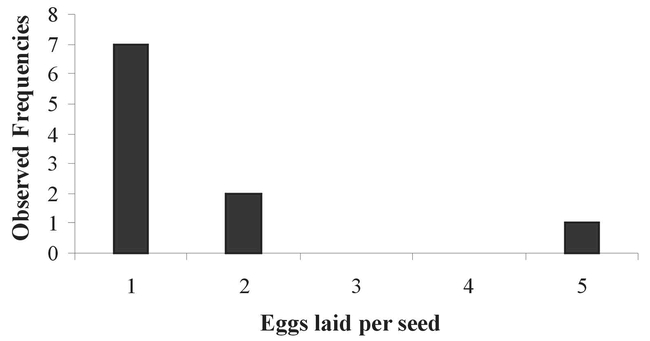
Egg-laying of Stator furcatus on seeds of Acacia caven. After a rainy spring the partially dehiscent pods permitted the egg-laying on seeds by S.furcatus.

**Figure 4.**
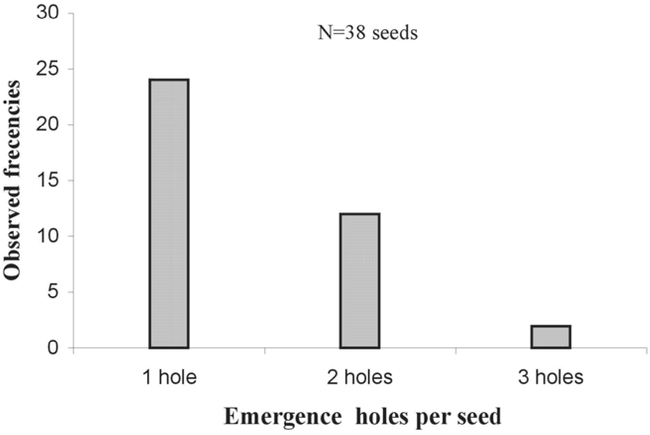
Emergence holes pierced per Acacia caven seed by Stator furcatus. From one to three S.furcatus could develop per seed.

Some pods (24%: 12/50) were contaminated by a second bruchid species: Stator furcatus, which is smaller than P. spinipes and lays 1 to 5 eggs on the integument of the seeds (Figure 3). In contrast to P. spinipes, 1 to 3 S. furcatus emergence holes were observed in seeds, indicating that more than one S. furcatus adult could develop per seed (Figure 4). P. spinipes egg-laying was also observed in the split open, partially dehiscent pods. The eggs were aggregated on the pod integument. Thus, the total level of bruchid attack was the sum of the emergence holes of the two bruchids (S. furcatus + P. spinipes) and those of parasitoids (smaller than those of bruchids). The presence of parasitoid emergence holes indicated the presence of a bruchid host that had been parasitized.

**Figure 5.**
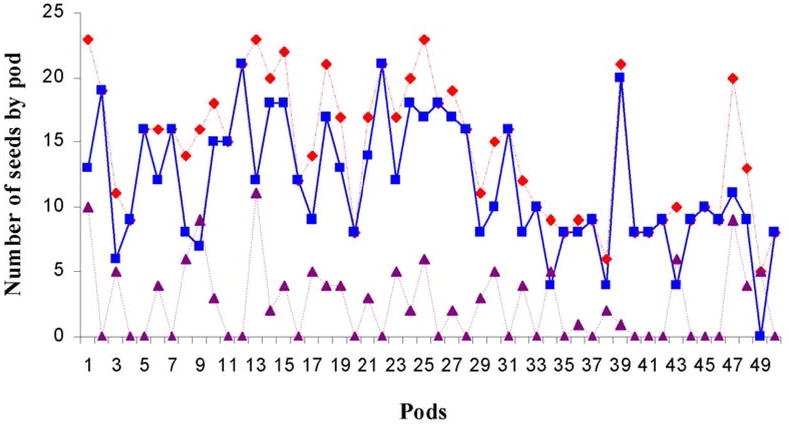
Change in seed quality per pod of Acacia caven. Fifty A. caven pods were opened, seeds attacked by Bruchid beetles were separated out and the ability of seeds to grow was evaluated. Total seeds (red line), seeds attacked by Bruchid beetles (blue line), live seeds (purple line).

Study of the change in seed quality indicated that the number of contaminated seeds was close to the total number of seeds (Figure 5), with 82% of the total seeds (587/717) attacked by the bruchid beetles. On average, there were 14.35 ± 1.45 seeds in a pod (mean ± SE), with 11.75 ± 1.40 contaminated by bruchid beetles (the difference observed was significant: Student test, t = 6.10; α = 0.05). The difference between the total number of seeds and the number of attacked seeds per pod corresponded to the seeds able to start growth (10.5%), and those that could not (7.5%).

### Parasitism of bruchids

The percentage of parasitized seeds per pod represented the ratio of parasitized seeds/total attacked seeds (Table 2). The parasitism of bruchid beetles differed between pods (χ^2^ calculated = 19.11: α = 0.01 χ^2^ddl 5 = 15.08). In fact, in 17 pods attacked by bruchid beetles there was no parasitism, while 2 out of 50 attacked pods were parasitized from 80 to 100% (Table 2).

**Table 2.**
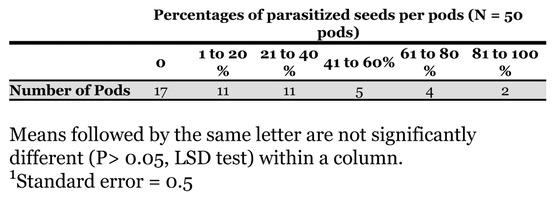
Pattern of parasitism in 50 Uruguayan pods attacked by Pseudopachymeria spinipes.

From all the pods attacked (Chilean + Uruguayan), 149 Monoksa dorsiplana females and 55 males emerged (Pteromalidae), and 384 females and 222 males of Horismenus *spp.* (Eulophidae). These parasitoids (total = 810) emerged from 285 seeds (with 1 P. spinipes host per seed), which could only be possible if the parasitoids had a gregarious larval development.

### Some life history traits of Monoksa dorsiplana

**Figure 6.**
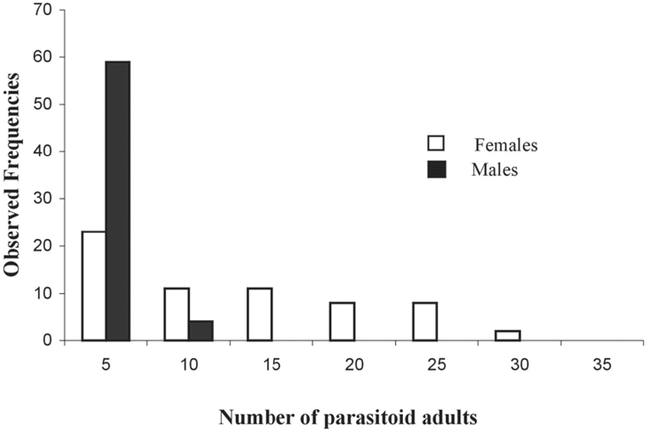
Total offspring of 63 egg-laying Monoska dorsiplana inseminated females. Each egg-laying female was provided with 1 host per day for 4 consecutive days.

**Figure 7.**
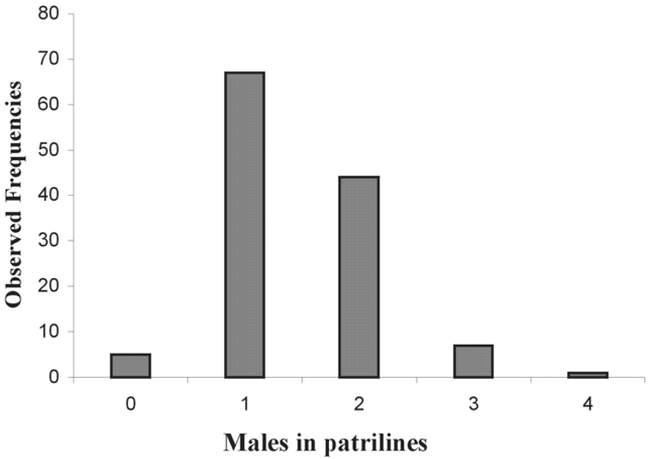
Distribution of male Monoksa dorsiplana in the patrilines of one egg-laying female.

**Figure 8.**
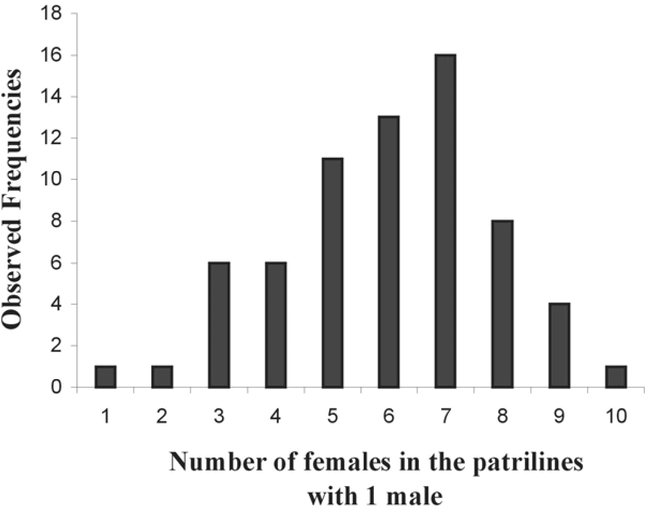
Females emerged per host parasitized by one egg-laying Monoksa dorsiplana female.

The substitution host (C. maculatus) was fully adopted by 70% of egg-laying females (44/63), allowing total development of M. dorsiplana. The distribution of male and female offspring showed that one egg-laying female (provided with one host per day for 4 consecutive days) could have a maximum of 34 daughters for 9 sons (Figure 6). The parasitoids emerging from one host allowed the most common associations to be studied (0, 1, 2 sons associated with 0, 1, 2 daughters etc.). The most common patriline of the offspring of one egg-laying female (provided with one host per day) was 1 male and 7 females (Figures 7 and 8).

## Discussion

The bruchid beetles attacking legume seeds, and the level of contamination, correlate to three patterns of pod dehiscence, classified as dehiscent, partially dehiscent and indehiscent (Johnson, 1981). According the pattern of dehiscence, the pods are attacked by three different guilds of bruchids, but this association of 'one pod dehiscence pattern/one species of bruchid beetle' is related to the different stages of pod or seed maturity (Johnson, 1981). In fact, all three bruchid guilds may attack one species of a partially dehiscent legume at different stages of pod or seed maturity, and indehiscent pods are sometimes tardily dehiscent after several months, the deterioration of the woody pods exposing the seeds (Johnson, 1981).

In Chile (region of Cauquenes), after a dry winter and spring (1997), all the persistent pods collected were indehiscent with highly sclerified walls. On the other hand, after a wet winter and spring, some persistent pods collected in Uruguay (2002) were partially dehiscent, inducing the deterioration of the woody pods, and consequently exposing the seeds. Such damaged pods allowed a diverse entomofauna to develop and/or to take refuge inside them. Hence, persistent pods of A caven provided a refuge for a variety of insect species, including two bruchid beetles (P. spinipes and S. furcatus), one scolytidae (Dendroctonus sp), lepidopterous larvae, ant colonies (Camponotus sp) and hymenopterous parasitoids (one oophagous parasitoid, Uscana espinae group *senex,* and larval-pupae parasitoids: M. dorsiplana, Horismenus spp.).

Of the indehiscent pods collected in Chile, with or without emergence holes, 68% were attacked by P. spinipes. However, the presence of emergence holes is not a reliable index of the intensity of bruchid beetle attack (Saiz et al., 1990). In fact, a maximum of 1 to 5 emergence holes were counted per pod, whereas 168 P. spinipes adults emerged from 27 pods with only 1 emergence hole. This observation indicates that the majority of bruchid beetles left the pod by the first pierced hole. As the construction of pods is multiseriate, each seed is enclosed in a lodge, so in order to leave the pod, the bruchid beetles must drill the walls of several lodges to reach the first pierced hole. This behavior could be explained by a positive photo-attractivity induced by light going through the first pierced hole.

In Uruguay, the semi-dehiscent pods already attacked by P. spinipes showed that 24% were infiltrated by a second species of bruchid beetle, S. furcatus, presumably due to the deterioration of their woody walls. Around 82% of the seeds of pods attacked by P. spinipes + S. furcatus were destroyed. In contrast to P. spinipes, more than one S. furcatus adult can develop per seed, and we observed up to 3 adults per seed.

The seeds of persistent pods of A. caven (which remained on the trees) were accessible to a guild of bruchid beetles after unfavorable climatic conditions that deteriorated the woody walls of the pods. The attack on seeds was most direct under these conditions, because they did not need to wait to lay their eggs for the transit of indehiscent pods through the digestive tract of mammals (Johnson 1981; Traveset 1990). For example, in Central America the indehiscent pods of A. farnesia are eaten by horses, deer and ctenosaur lizards, and the seeds that pass intact in the feces are attacked by S. vachelliae (Traveset 1990).

These two species, P. spinipes and S. furcatus, constitute a significant stock of adults able to infect legumes. The P. spinipes adults emerging from persistent pods lay their eggs from January to March on the new green pods which the neonatal larvae are able to penetrate more easily than sclerified pods (Saiz et al., 1980).

Two native Trichogrammatidae were recently found as oophagous parasitoids of bruchid beetle eggs: Uscana chiliensis (Pintureau and Gering) on Bruchus pisorum, and Uscana espinae (Pintureau and Gering) on P. spinipes (Pintureau et al., 1999). In addition, one Pteromalidae (M. dorsiplana) and two Eulophidae (two species of Horismenus *spp.*) emerged from seeds of the persistent pods of A. caven attacked by P. spinipes. These persistent pods appear to be a refuge for bruchid parasitoids. This new information should facilitate the study of native parasitoids of South America for biological control of Bruchidae.

The species Horismenus *spp.* represents 59.1% of the parasitoid community of Bruchidae Acanthoscelides sp. and Zabrotes *sp.* (Hansson et al., 2004). The genus Horismenus is predominantly a New World group, with its main distribution in the neo-tropical region. Currently there are 53 known species in the Americas and one species in Europe. The species are parasitoids or hyperparsitoids of Coleoptera, Diptera and Lepidoptera larvae. The majority of the species have so far not been described, and the identities of many of the 50 or so species that have been described are unclear (Hansson et al., 2004).

The Pteromalidae M. dorsiplana was first described in 1990 (Boucek, 1990). This is the first time that this parasitoid has been found in South America on seeds of partially dehiscent persistent pods of A. caven attacked by P. spinipes. As only one bruchid beetle can develop in a seed, we conclude that this species is a gregarious larval ectoparasitoïd (proportion of females: 0.73). In contrast to Horismenus *spp.,* M. dorsiplana was reared on a substitution host (C. maculatus) without any specific selection, since 70% of females produced offspring. The patriline of M. dorsiplana was generally composed of 1 son + 7 daughters.

The persistent indehiscent pods of A. caven that become partially-dehiscent under unfavorable climatic conditions (rainy season during winter and spring), provide a refuge for a large entomofauna. In addition to the bruchid beetle of Acacia *sp.* (P. spinipes), partial dehiscence may be a favorable condition for attack by a second bruchid (S. furcatus), which normally attacks seeds fallen on the substrate. This bruchid community may provide a reserve of parasitoids that could be used for biological control of Bruchidae.
